# Supplementary material for: Rapid Minimum Inhibitory Concentration (MIC) Analysis Using Lyophilized Reagent Beads in a Novel Multiphase, Single-Vessel Assay
Source: Antibiotics (Basel). 2023 Nov 19;12(11):1641. doi: 10.3390/antibiotics12111641 (PMC10669664; doi:10.3390/antibiotics12111641)
Supplement: Supplementary file 1 [file antibiotics-12-01641-s001.zip › antibiotics-2673091-supplementary.pdf]

antibiotics

MDPI

Supplementary Materials

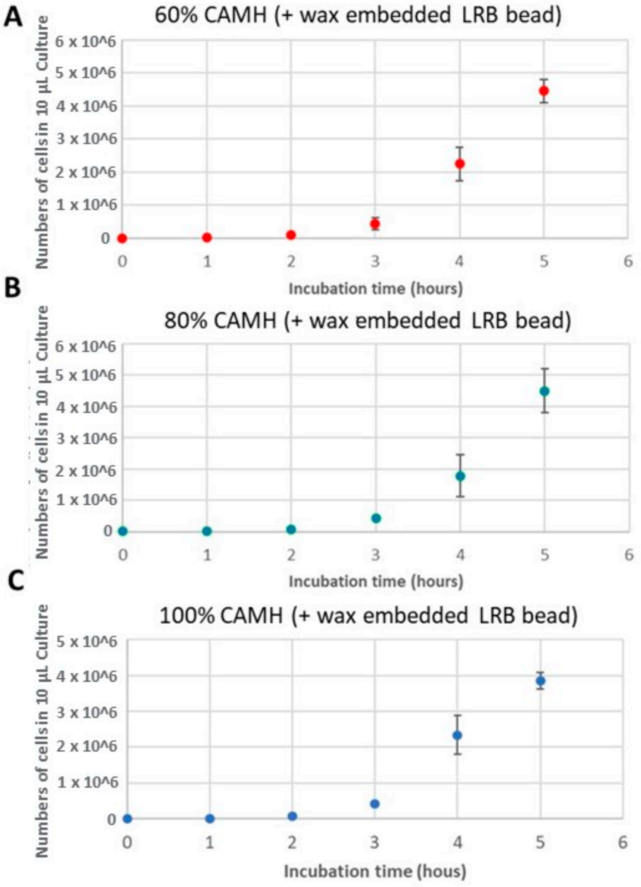

Figure S1. Test of bacteria growth in multi-phase wax/lyophilized reagent bead (LRB) environment. Growth analysis of E. coli (ATCC 25922) in 60% (A), 80% (B), or 100% (C) cation-adjusted Muller-Hinton (CAMH) growth buffer with a drug-free LRB (lyophilized reagent bead) beads added to the cultures to test compatibility with growth assays. Growth was performed using a 5000-cell seeding density in 0.1 mL PCR tubes at 37 °C with triplicate tubes for each data point. At each time point, bacteria were removed and plated on TSA plates for colony counting (plates incubated at 37 °C). The bead components/materials did not inhibit growth at any of the tested CAMH growth buffer concentrations over 6 hours, demonstrating compatibility with growth during the short incubations used herein prior to PCR analysis.

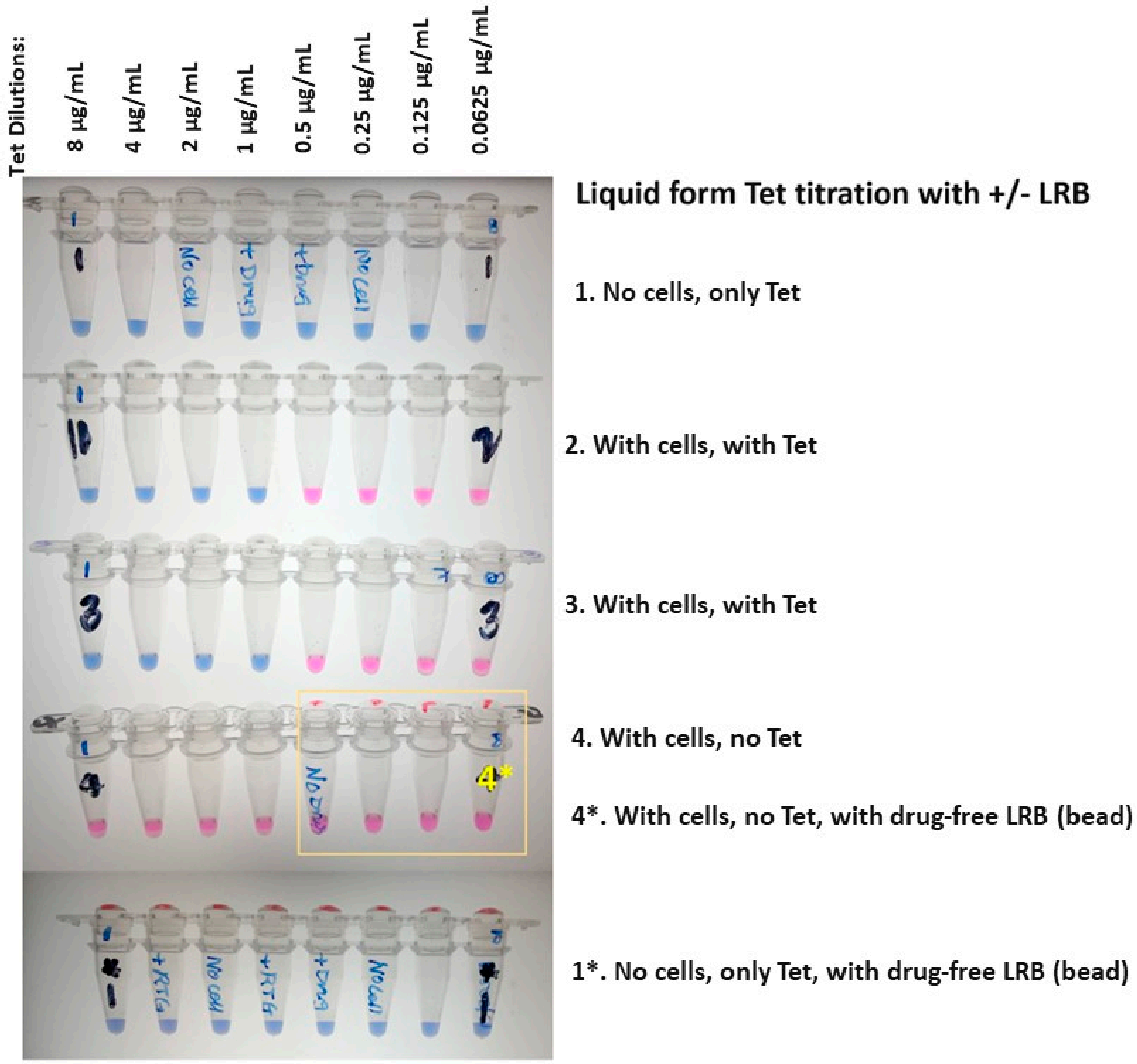

Figure S2. Comparing antimicrobial (tetracycline) mean inhibitory concentration (MIC) outcomes with and without the presence of lyophilized bead reagents (in liquid form). Images of alamarBlue®/resazurin assay performed in tubes following CLSI guidelines using E. coli (ATCC#25922) at a seeding density of 500 CFU/µL. Concentrations of tetracycline hydrochloride (Tet (tetracycline); Sigma (St. Louis, MO, USA) T3383-25G) were diluted to concentrations of 8–0.0625 ug/mL in CAMH (cation-adjusted Mullen-Hinton) growth buffer. Cells were grown at 37 °C for 16 hours with no agitation. The alamarBlue assay readout is blue for sterile (no bacteria), purple/blue for some bacteria growth initiated, and pink for full growth conditions. The Tet concentrations used in each tube/condition are shown on the top. Each row of tubes from top to bottom had 1. No bacteria cells added (Tet-only control); 2. Bacteria added in all tubes across each drug concentration with growth shown at the 0.0625, 0.125, 0.25, and 0.5 ug/mL levels; 3. Replicate set with bacteria again added in all tubes across each drug concentration with growth shown at the 0.0625, 0.125, 0.25, and 0.5 ug/mL levels; 4. (left side; 4 tubes) Bacteria added with no Tet (no-drug control); 4*. (right side; 4 tubes) Bacteria added with no Tet and with the addition of an LRB (lyophilized reagent bead; containing no drug, just LRB components); and 1* (bottom row) No bacteria/cell control with Tet and drug-free LRB bead in each tube.

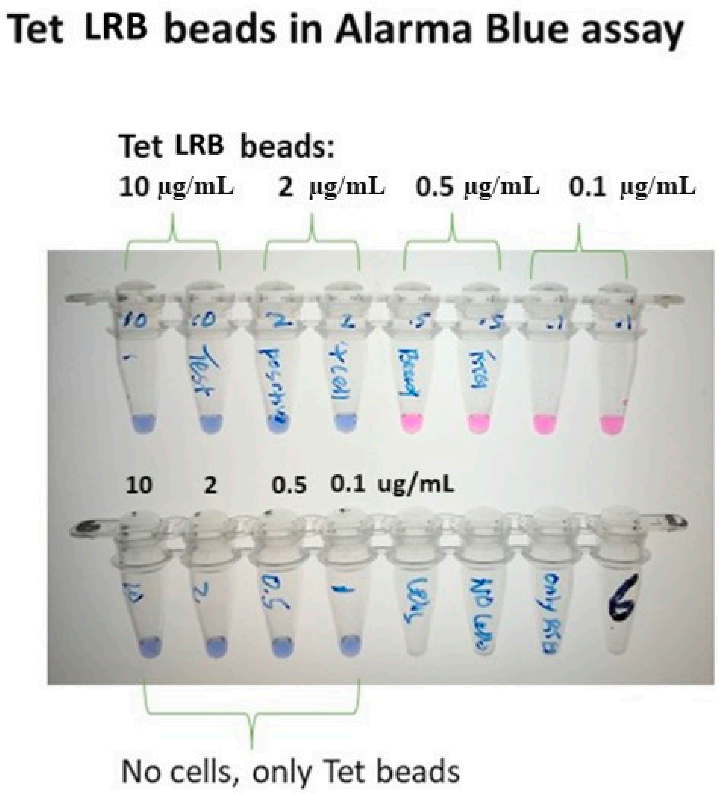

Figure S3. Comparing antimicrobial (tetracycline) mean inhibitory concentration (MIC) outcomes with and without the presence of lyophilized beads. Images of alamarBlue®/resazurin assay performed in tubes following CLSI guidelines using E. coli (ATCC#25922) at a seeding density of 500 CFU/µL. Tetracycline (Tet) lyophilized reagent beads (LRB) were made as described in method with final Tet concentrations of 0.1, 0.5, 2, or 10 ug/mL and added to the growth tubes prior to loading sample/bacteria. Cells were grown at 37 °C for 16 hours with no agitation. The alamarBlue assay readout is blue for sterile (no bacteria), purple/blue for some bacteria growth initiated, and pink for full growth conditions. In the top row, bacteria were added to all tubes/drug concentrations and growth occurred in all tubes with Tet LRB containing 0.5 ug/mL or lower. In the bottom row, the 4 left tubes contained 10, 2, 0.5, and 0.1 ug/mL Tet beads but no bacteria, while the 4 right tubes contained no alamarBlue.

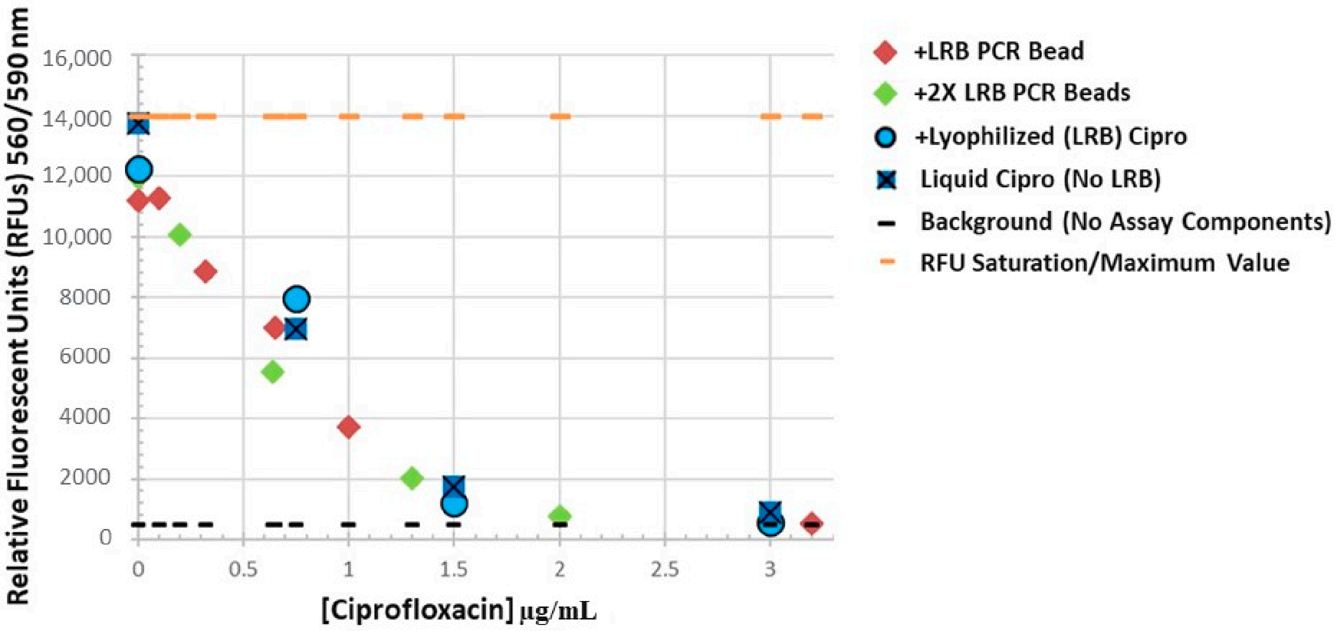

Figure S4. Comparing antimicrobial (ciprofloxacin) mean inhibitory concentration (MIC) outcomes with and without the presence of lyophilized beads and/or lyophilized PCR reagents. AlamarBlue®/resazurin control assays were performed in tubes with E. coli (ATCC#25922) at a seeding density of 10^3 CFU and using 20-hour growth period. Ciprofloxacin (cipro) at multiple concentrations was added either as a lyophilized reagent bead (LRB; blue dot) or liquid form (all others). These longer growth assays were performed to ensure that LRB formulations of the antimicrobial functioned and of the standard liquid form. For two datasets, liquid cipro was added to growth wells along with 1 (red triangle) or 2 (green triangle) blank LRB beads without cipro in the bead. For an additional dataset, liquid cipro was added to the growth wells with no additional LRB reagents (liquid or lyophilized). The background levels for wells with no assay components/buffer alone (black dotted line) and the saturation/maximum limit (orange dotted line) are shown. An additional outcome of this figure was demonstration of LRB functionality with LRBs manufactured and stored for one month at room temperature prior to use (light blue dots). As with the commercial PCR LRBs[25], the antimicrobial LRBs are stored either in low-humidity environments or sealed plastic cartridges containing desiccant and humidity-sensing cards.

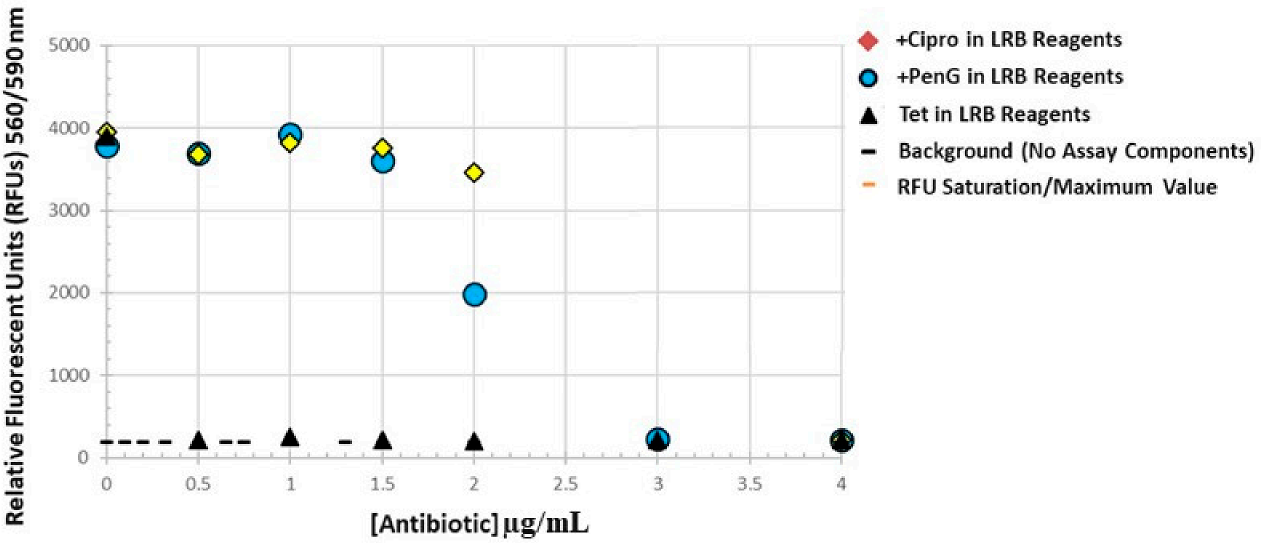

Figure S5. Comparing antimicrobial (ciprofloxacin, tetracycline, or Penicillin G) mean inhibitory concentration (MIC) outcomes in lyophilized beads format. AlamarBlue®/resazurin assay performed in plates with E. coli (ATCC#25922) at a seeding density of 10^3 CFU for 20-hour growth periods. Ciprofloxacin (cipro; red triangles), Pen G (blue dots), and tetracycline (Tet; black triangles) were added to the growth buffer along with liquid LRB reagents at multiple concentrations from 0 to 4 µg/mL. The background levels for wells with no assay components/buffer only (black dotted line) and the saturation/maximum limit (orange dotted line) are shown. The inhibitory concentrations observed were 2.5 µg/mL, 3.5 µg/mL, and <0.5 mL µg/mL.

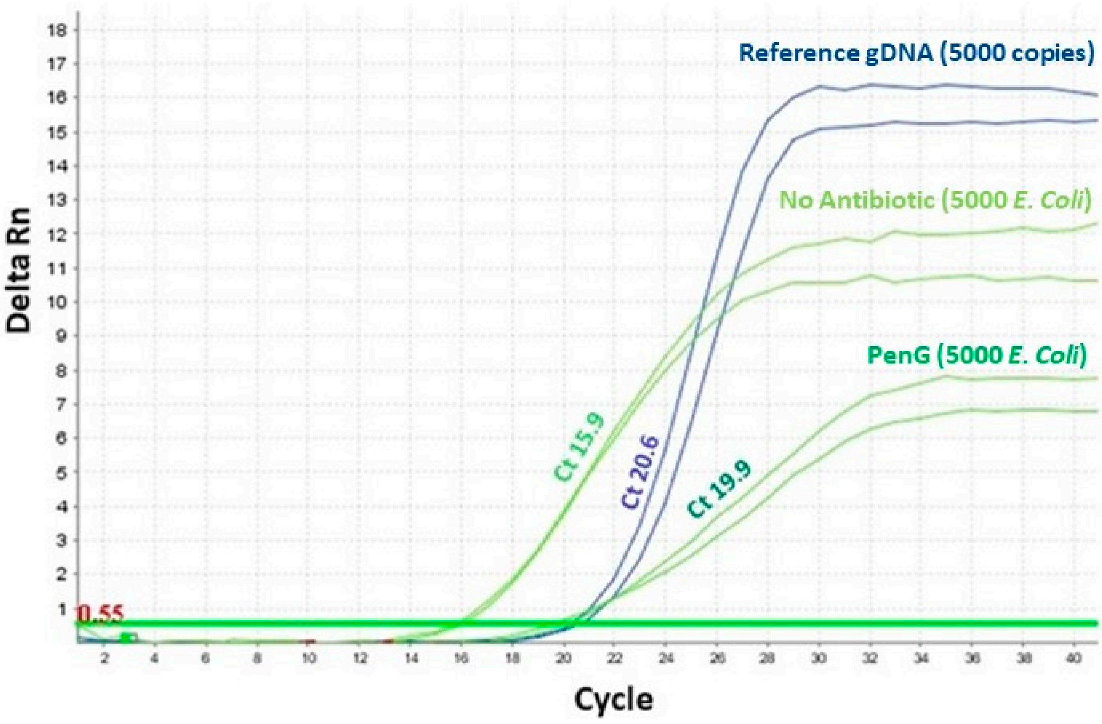

Figure S6. AST measurements made using the multi-phase reaction chamber/tubes using Penicillin G. Multiphase reaction chambers with wax-covered PCR LRBs were tested using either reference gDNA or E. coli added above the wax barrier (in 1X CAMH growth broth) and incubated for 2 hours at 37 °C prior to melting the wax and initiating PCR. gDNA was added at 5000 copies, while E. coli was added at 5000 CFU. Therefore, Ct values for chambers with no growth/bacteria doubling should be similar, while chambers that demonstrate growth/bacteria doubling initiate PCR with a higher concentration of starting template and therefore demonstrate a lower Ct. Each curve represents the corresponding PCR results from an individual chamber. Chambers containing E. coli either contained no antibiotic (light green) or 10 µg/mL PenG (dark green) and were compared to the gDNA-containing chambers with no antibiotic added (dark blue).

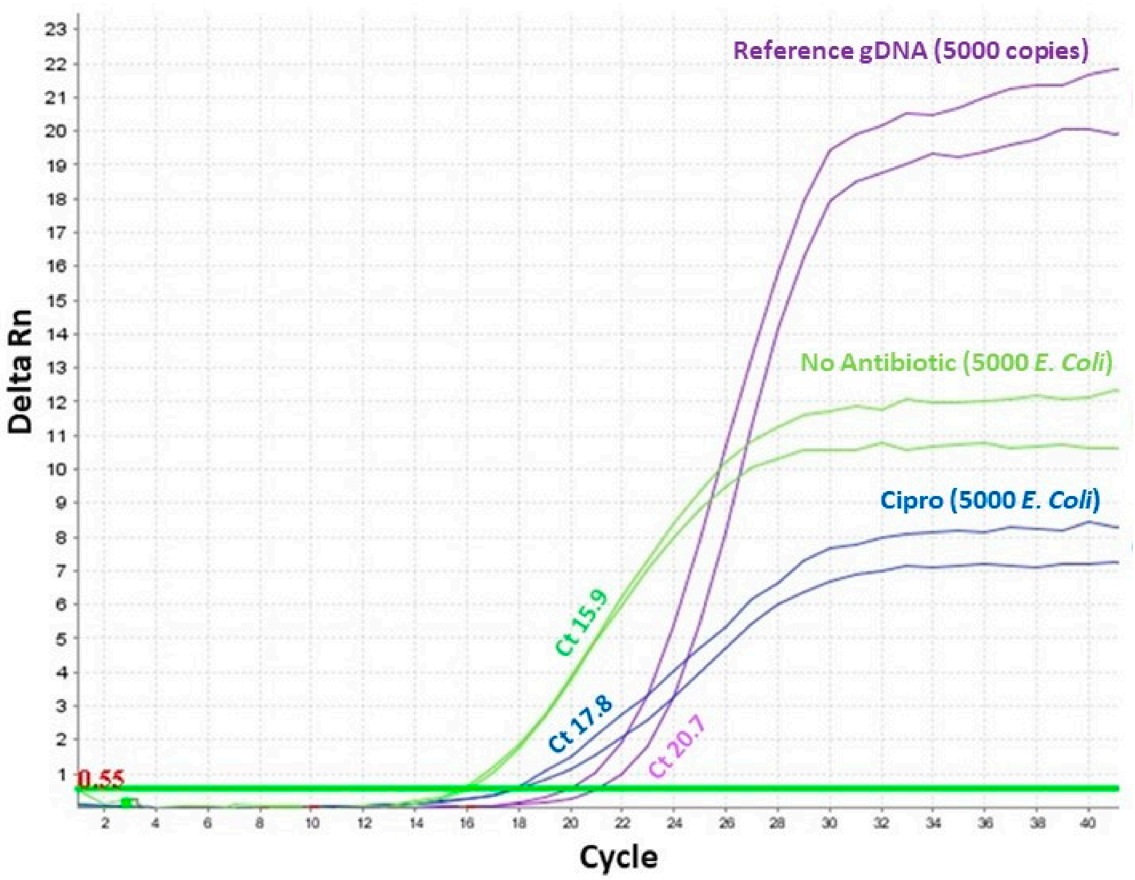

Figure S7. AST measurements made using the multi-phase reaction chamber/tubes using Ciprofloxacin. Multiphase reaction chambers with wax-covered PCR LRB were tested using either reference gDNA or E. coli added above the wax barrier (in 1X CAMH growth broth) and incubated for 2 hours at 37 °C prior to melting the wax and initiating PCR. gDNA was added at 5000 copies, while E. coli was added at 5000 CFU. Therefore, Ct values for chambers with no growth/bacteria doubling should be similar, while chambers that demonstrate growth/bacteria doubling initiate PCR with a higher concentration of starting template and therefore demonstrate a lower Ct. Each curve represents the corresponding PCR results from an individual chamber. Chambers containing E. coli either contained no antibiotic (light green) or 10 µg/mL Cipro (dark blue) and were compared to the gDNA-containing chambers with no antibiotic added (purple).

(A)

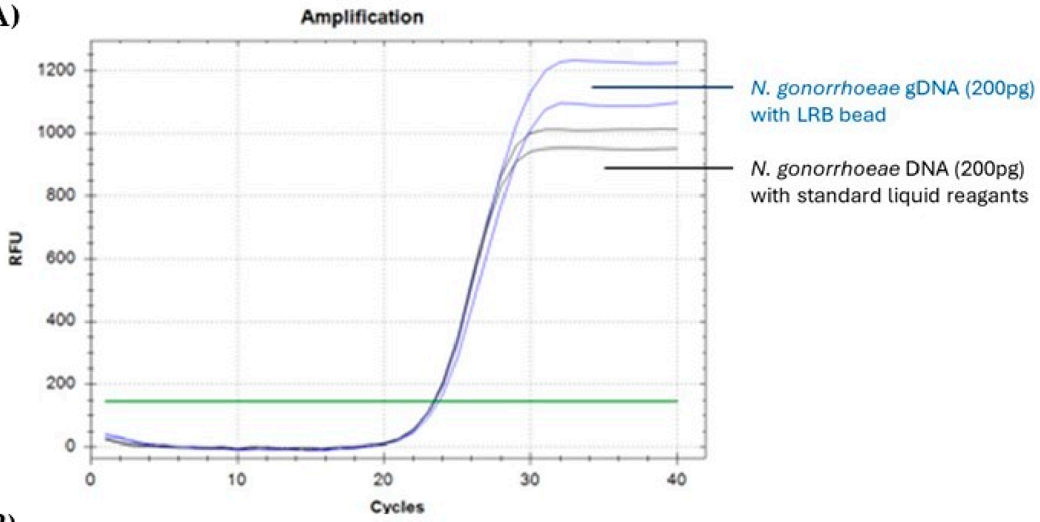

(B)

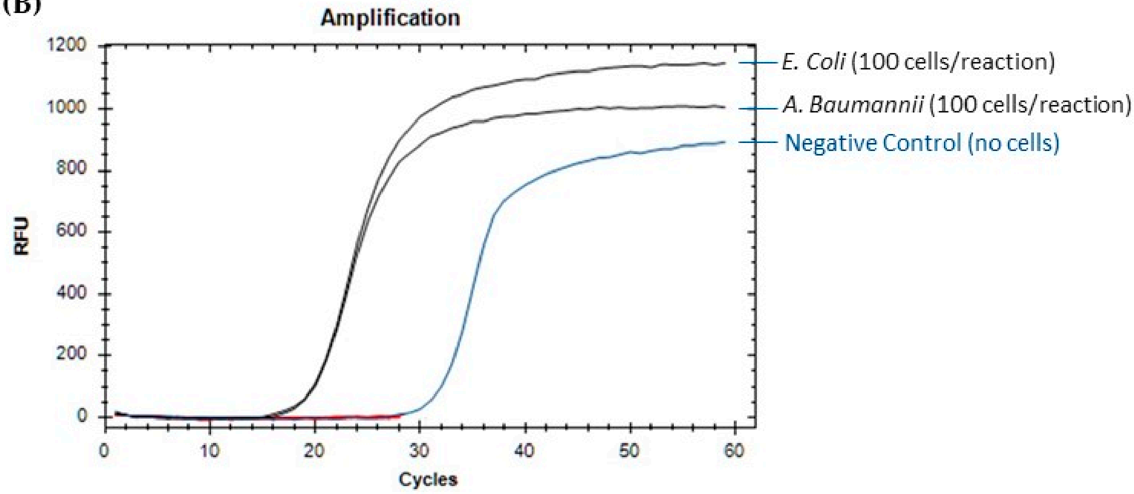

Figure S8. Use of lyophilized bead PCR reagents with multiple pathogen and/or DNA sample types. While use of the LRB formulation for antibiotic storage is novel, LRB formulations for PCR reagents is available as a commercial product [23],, and manufactured and used for a wide variety of applications, including microbial detection and analysis. Most of the work in this manuscript utilized E. coli-specific primers, thus rendering the beads E. coli-specific. While it is beyond the scope of this manuscript to replicate use of the LRB bead/lyophilized PCR reagents across many pathogens and microbial species, Figure S8 demonstrates use of both a second pathogen primer,[38], and a universal 16S rDNA primer set [39[, , within either an LRB (**A**) or an LRB liquid component (**B**; using alternative PCR enzyme; SsoAdvanced; Biorad; Hercules, CA, USA) PCR mixture. The beads are capable of being loaded with a wide variety of PCR enzymes, primer sets, and buffer/salt mixtures, thus making them compatible across pathogen types (i.e., bacteria or virus) or species.

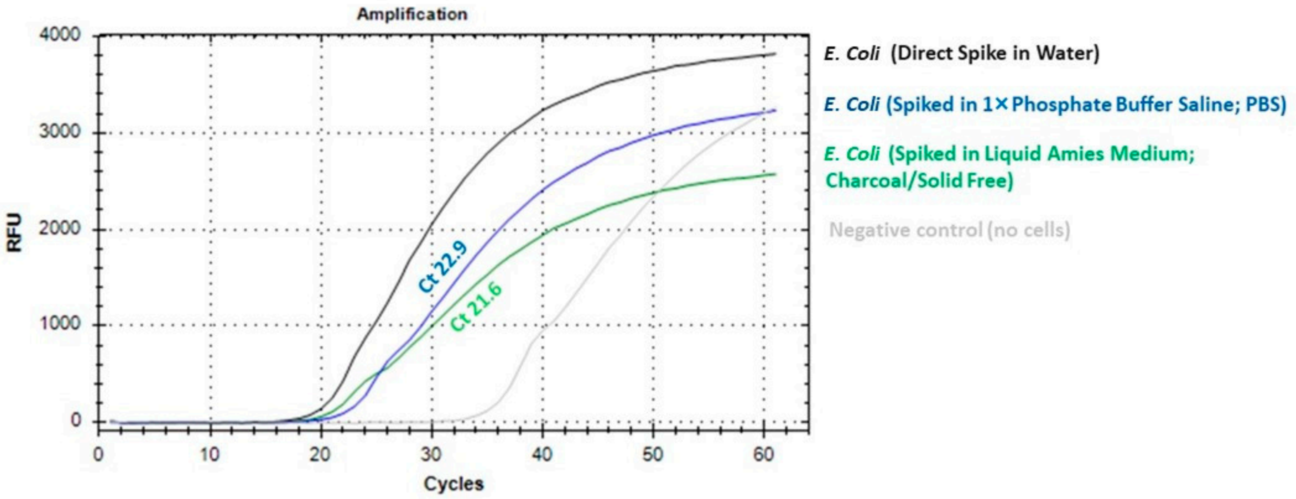

Figure S9. Use of lyophilized bead PCR reagents with multiple buffer and/or sample input types. Reactions using a PCR LRB were set up using either water, phosphate-buffered saline, or a swab transport medium (liquid amies medium). As shown above (using CAMH bacteria growth medium and 100 spiked bacteria), PCR using the LRB is viable in high-salt-sample environments (Ct in PBS (22.9) and amies (21.6) versus negative/no template controls (NTC); 100 bacteria spiked), including the PBS and amies medium (two commonly used transport buffers for infectious disease/microbial testing). As reported by our group previously, swab samples are a common transport medium for nasal and sexually transmitted disease (STD) sample types [29], , and initial applications of the two-phase reaction system reported herein remain compatible with the reagents required for transport and sampling in these systems.

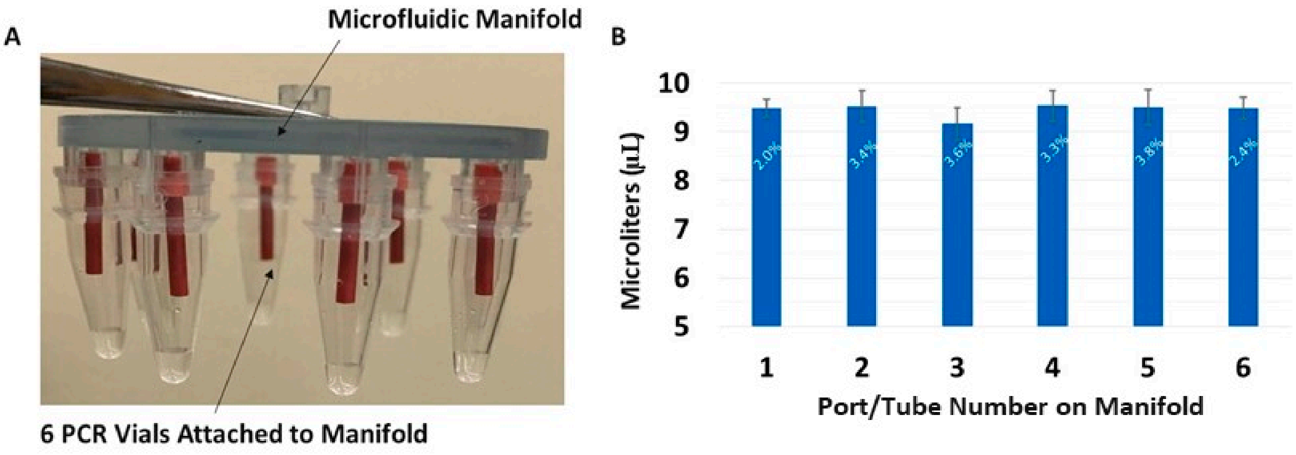

Figure S10. Measuring Coefficient of Variance of fluid output in different ports of the microfluidic sample splitting manifold. (A) The manifold was used in control tests to ensure that liquid distribution and sample splitting were even across all 6 PCR vials. (**B)** Fluid distribution across the 6 vials was measured using a manifold with three separate loadings (after drying and reuse and weighing the increase in weight in each vial after sample addition). The coefficient of variation (shown as % text within the data bar) was under 4.0% for all manifold lines/vials with an average of 3.1% variation across the device.
